# A novel phosphoester-based cationic co-polymer nanocarrier delivers chimeric antigen receptor plasmid and exhibits anti-tumor effect†

**DOI:** 10.1039/c8ra02133c

**Published:** 2018-04-19

**Authors:** Jing Fan, Qianjun He, Zhaokui Jin, Wei Chen, Weiren Huang

**Affiliations:** Key Laboratory of Medical Reprogramming Technology, Shenzhen Second People's Hospital, First Affiliated Hospital of Shenzhen University Shenzhen 518039 China pony8980@163.com jessie_chenwei@163.com; Sun Yat-sen University Cancer Center, State Key Laboratory of Oncology in South China Guangzhou 510060 China; Guangdong Key Laboratory for Biomedical Measurements and Ultrasound Imaging, School of Biomedical Engineering, Health Science Center, Shenzhen University No. 3688 Nanhai Road, Nanshan District Shenzhen 518060 China

## Abstract

Chimeric antigen receptor T cells (CAR-T cells) targeting of CD19 antigen has been proven to be effective and successful in B cell acute lymphoblastic leukemia. The traditional CAR delivery systems have several problems such as poor biosafety, low loading capacity, and low transfection efficiency. Utilization of nanocarriers for CAR delivery offers new possibilities for CAR-T treatment. In the present study, an anti-CD19 CAR expression lentivirus plasmid was constructed for CAR delivery and immunotherapy. In addition, a three-segment amphiphilic co-polymer, methoxy polyethylene glycol-branched polyethyleneimine-poly(2-ethylbutyl phospholane) (mPEG-bPEI-PEBP) was synthesized *via* click reaction as the carrier with cationic PEI, capable of delivering the CAR and packaging plasmids to co-transfect Jurkat cells and undergo expression. The PEBP and mPEG parts of the co-polymer provide hydrophobic and hydrophilic interfaces and lead to the co-polymer self-assembly into micelles in water and encapsulation of the DNA plasmids. The mPEG-bPEI-PEBP-DNA composites with different N/P ratios were incubated with the CD19 overexpression K562 cells to identify the CAR functions. The obtained CAR-Jurkat cells had the ability to secrete interferon-γ and interleukin-2. The cytotoxic effects to CD19-K562 cells suggest that the induced CAR-Jurkat cells have an excellent targeted antitumor activity.

## Abstract 2

A three-segment amphiphilic co-polymer mPEG-bPEI-PEBP was synthesized as the nanocarrier with cationic PEI, capable of delivering the CAR and packaging plasmids into Jurkat cells to generate the CAR-T cells for anti-CD19 immunotherapy study.

## Graphical abstract

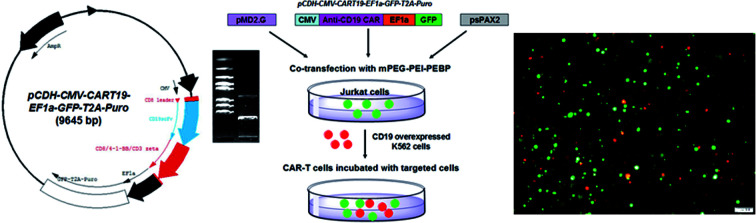

## Introduction

Traditional radiotherapy and chemotherapy, the main modalities of cancer treatment, have numerous limitations including toxicity and lack of specific targeting. Various modifications of tumor immunotherapy have been developed in recent years following discovery of the underlying immune mechanisms.^1^ Among them, chimeric antigen receptor (CAR) T cell therapy has acquired considerable attention. CAR-T cells express surface chimeric receptors that can be used for tumor-specific targeting, and act as immune activators and stimulators of T cells. After reinfusion *in vivo*, CAR-T cells are capable of amplification, establishing immune memory and providing continuous surveillance to treat local or metastatic cancer cells.^2–4^ CAR-T therapy is characterized by excellent therapeutic results in hematologic tumors and melanoma.^5^ The first anti-CD19 CAR-T therapy for acute lymphoblastic leukemia was approved by the U.S. Food and Drug Administration in 2017. However, traditional strategies for CAR delivery into T cells such as the adeno-associated virus,^6^ lentivirus and other biological carriers,^7^ have several shortcomings. These include biosafety issues, and low loading capacity and transfection efficiency.^8–10^ In this regard, nanomaterials may have advantages as CAR plasmid carriers.^11^ Their excellent capacity for chemical modification means that nanocarriers can be multi-functionalized by modifying different ligands. For example, after enhancing their ability to bind the target ligand or responsive groups, nanocarriers can acquire a bio-targeting function. Surface modifications can improve water-solubility of nanocomposites and extend blood circulation time. Some features of nanoscale materials such as enhanced permeability and retention effect (EPR) are beneficial for targeting solid tumors *in vivo*.^12–16^

**Fig. 1:**
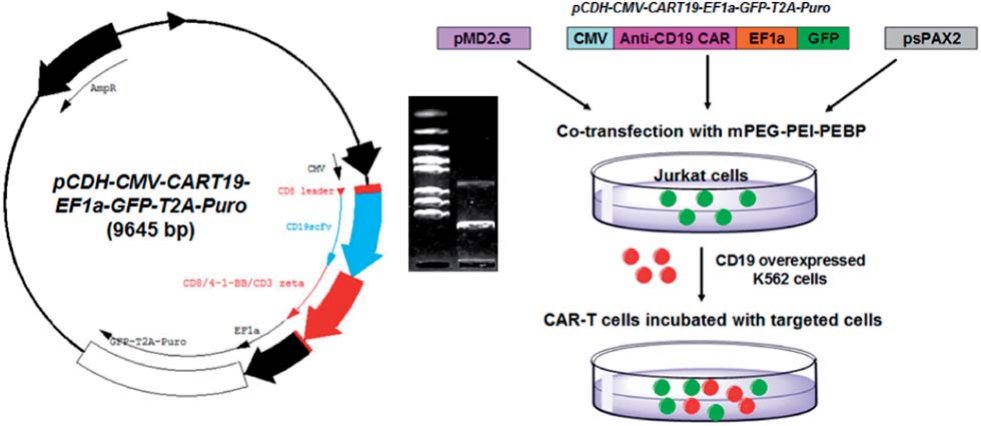
The structural diagram of anti-CD19 CAR lentivirus plasmid (pCDH-CMV-CART19-EF1α-GFP-T2A-Puro, left) and its identification by enzyme digestion (the digital photo); the CAR plasmid and lentiviral packaging plasmid pMD2.G and psPAX2 were co-transfected with mPEG-PEI-PEBP nanocarriers into Jurkat cells to generate the CAR-T cells for anti-CD19 (K562 cells) immunotherapy study (right).

The polyethyleneimine (PEI) polymer is rich in amine groups and displays a high cationic charge density, which allows ionic interactions with the phosphate backbone of DNA.^17^ The PEI-DNA complex can pass through the cell membrane anionic charge to deliver loaded DNA into the cell where it can perform its biological functions.^18–23^ We have demonstrated previously that the cytotoxicity and side effects of branched polyethyleneimine (bPEI) can be decreased by wrapping the molecule in amphiphilic block polymers. This modification does not affect the electropositivity of PEI.^24–26^ Therefore, the objective of this study was to synthesize an amphiphilic co-polymer capable of loading the anti-CD19 lentivirus plasmid (pCDH-CMV-CART19-EF1α-GFP-T2A-Puro) by electrostatic force. The bPEI was modified with 2-azidoacetic acid to introduce an azido group. It was then subjected to an azide–alkyne click reaction with biocompatible alkyne-terminated monomethoxy polyethylene glycol (mPEG-alkyne) and alkyne-terminated poly(2-ethylbutyl phospholane) (alkyne-PEBP) polymer to generate the three-segment mPEG-bPEI-PEBP amphiphilic co-polymer.^26^ The synthesized co-polymer could self-assemble into a micelle with a hydrophobic PEBP core and a hydrophilic PEG shell. The electropositive bPEI with abundant amino terminal could be loaded with anti-CD19 plasmid or other functional plasmids for delivering into human immortalized T lymphocyte cell line Jurkat cells.^27^ After degradation, the loaded CAR and co-stimulatory elements were released and expressed inside CD19-overexpressing target cells. The target cells were derived from human myelogenous leukemia k562 cells,^28^ and designated as CD19-K562 cells. These cells were exposed to the anti-CD19 CAR-Jurkat cells and the resultant immunological effects were studied (Fig. 1).

## Experimental section

### Preparation of anti-human CD19 CAR lentivirus vectors

The anti-human CD19 (anti-CD19) CAR lentivirus core plasmid and packaging plasmids were designed and constructed in our laboratory as reported previously.^3,29^ The anti-CD19 scFv domain involved in T cell activation together with the co-stimulatory domains of 4-1BB and CD3ζ formed the anti-CD19 CAR functional domains. In the downstream region of CAR, a DNA sequence coding for an EF1α promoter-driven green fluorescent protein (GFP) was introduced to trace plasmid expression. The constructed CAR plasmid was identified by enzyme digestion (XbaI and EcoRI, Thermo Co., Ltd) and sequencing. Commercially available lentiviral packaging plasmids, psPAX2 and pMD2.G, were used for virus production.

### Construction of human CD19-overexpressing K562 cell strain

Stable clones expressing coding sequences for human CD19 and red fluorescent protein (RFP) were obtained by co-transfecting the three lentiviral vectors at a total weight of 24 μg (pCD19-RFP : psPAX2 : pMD2.G = 4 : 3 : 1, w/w) per 90 mm dish seeded with 10^6^ of 293T cells in DMEM containing 10% fetal bovine serum (Life Technologies Co., Ltd). After 48 h and 72 h, the medium (about 10 mL) was collected in 15 mL tube, and 2.5 mL of lentivirus concentration solution (Shanghai Yeasen Biotechnology Co., Ltd) was added and incubated overnight to precipitate virus particles. The collected live viruses were used to infect K562 cells. The viruses collected from one dish were used to transfect 10^5^ of K562 cells in a 6-well plate (Corning Inc.) containing RPMI 1640 supplemented with 10% fetal bovine serum. Colonies arose after 7–10 days, and stable clones were transferred to 24-well plates. In this process, RFP was used as a marker to monitor CD19 expression. The stable transfected clones of CD19-K562 cells were purified by fluorescence-activated cell sorting (BD FACSAria II), and the collected positive cells were incubated for use as target cells in subsequent experiments.

### Synthesis of bPEI-azide

The bPEI-azide was synthesized according to our published method.^26^ Briefly, 100 mg of branched PEI (Sigma-Aldrich, MW = 2000) was mixed with 18 mg of 2-azidoacetic acid (Sigma-Aldrich), 280 mg of 2-(1*H*-benzotriazol-1-yl)-1,1,3,3-tetramethyluronium hexafluorophosphate (HBTU, Sigma-Aldrich), 120 μL of triethylamine (TEA, Sigma-Aldrich) and 10 mL of dimethyl formamide (DMF, Sigma-Aldrich). The reaction mixture was incubated overnight to allow occurrence of the amidation reaction between the amine group of PEI and carboxyl group of 2-azidoacetic acid. The solution was then kept in a 2000 Da MW dialysis membrane (Spectra/Por) and dialyzed against water. The dialysate was replaced five times to remove unreacted reagents. The purified product was freeze–dried and stored at −20 °C.

### Synthesis of PEBP-alkyne

**Fig. 2:**
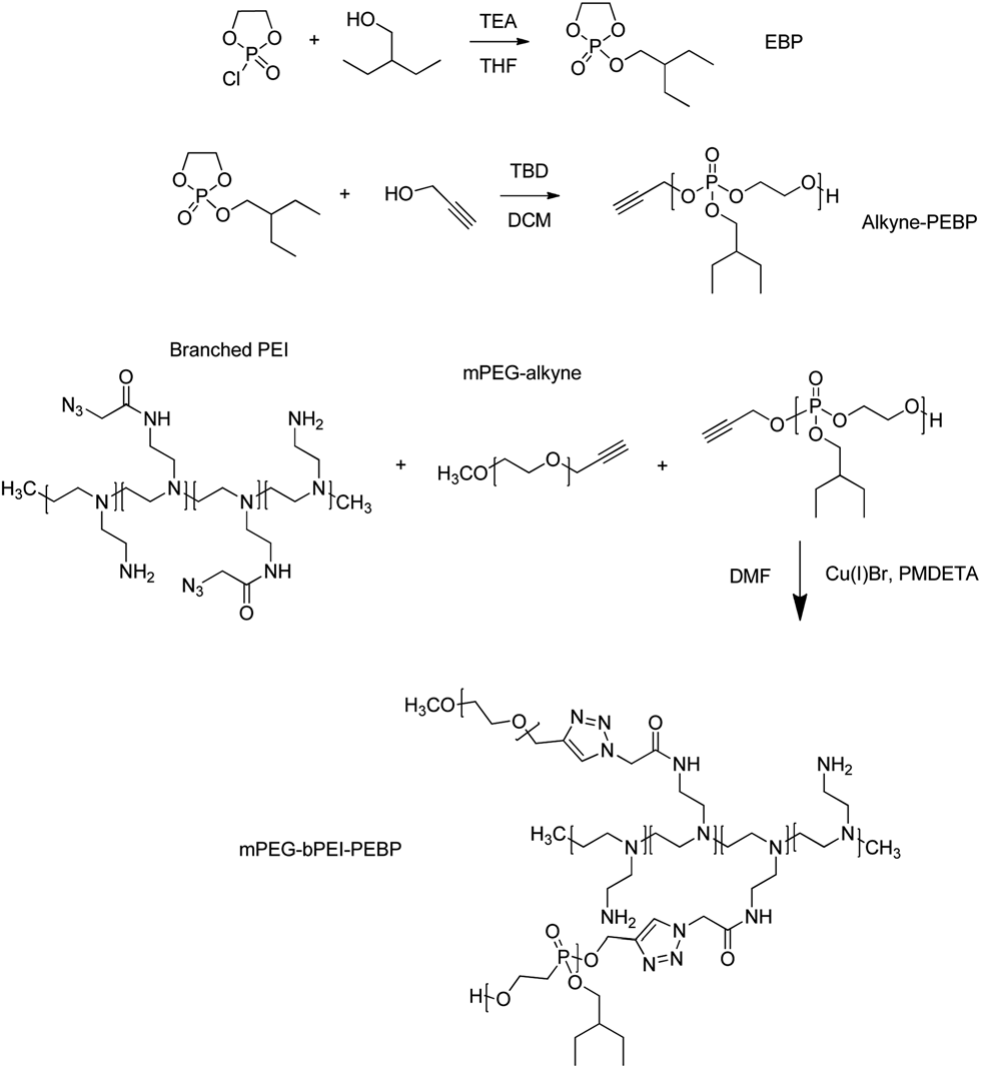
Synthesis of 2-ethylbutyl phospholane (EBP), alkyne-poly(2-ethylbutyl phospholane) (alkyne-PEBP), and mPEG-bPEI-PEBP co-polymer. TEA, triethylamine; THF, tetrahydrofuran; DMAP, dimethylaminopyridine; TBD, 1,5,7-triazabicyclo[4.4.0]dec-5-ene; DCM, dichloromethane; PEI, polyethyleneimine; mPEG, monomethoxypolyethylene glycol; DMF, dimethyl formamide; PMDETA, *N*,*N*,*N*′,*N*′′,*N*′′-pentamethyldiethylenetriamine; mPEG-bPEI-PEBP, poly(ethylene glycol)-arsenic-retinoic acid.

A solution of 2-chloro-1,3,2-dioxaphospholane 2-oxide (COP) (1 g, 7 mmol) in 15 mL of anhydrous tetrahydrofuran (THF, Sigma-Aldrich) was added dropwise, under constant stirring, to a solution of 2-ethyl-1-butanol (0.8 g, 7.8 mmol, Sigma-Aldrich) and triethylamine (0.79 g, 7.8 mmol, Sigma-Aldrich) in 100 mL of THF at 0 °C. The reaction mixture was stirred for a further 12 h in an ice bath (Fig. 2). After complete conversion of COP, as confirmed by thin layer chromatography, the reaction mixture was filtered and the filtrate was concentrated. The concentrated filtrate was distilled at 118–121 °C under reduced pressure (0.4 mmHg) to obtain a colorless viscous liquid. The yield was 0.8 g and the purity was 73% that determined by ^1^H NMR and ^31^P NMR in CDCl_3_.

The synthesized EBP (730 mg, 3.5 mmol) and 2-propynyl alcohol (6.5 mg, 0.12 mmol, Sigma-Aldrich) were dissolved in 5 mL anhydrous dichloromethane (DCM, Sigma-Aldrich). The catalyst, 25 mg of 1,5,7-triazabicyclo[4.4.0]dec-5-ene (TBD, 0.18 mmol, Sigma-Aldrich) dissolved in 1.0 mL DCM, was then added *via* syringe under nitrogen gas atmosphere and at 0 °C (Fig. 2). After stirring for 10 min, excess acetic acid was added to quench the reaction. The solution was then added dropwise into 50 mL petroleum ether to precipitate the target product PEBP. Subsequently, the mixture was centrifuged at 9000 rpm for 5 min to remove the supernatant and impurities. This purification step was repeated three times. Residual solvent was removed under vacuum. The obtained colorless transparent liquid was dissolved in CDCl_3_ for ^1^H NMR characterization.

### Synthesis of mPEG-bPEI-PEBP

Prepared bPEI-azide (MW = 2300) 100 mg along with 220 mg of mPEG-alkyne (MW = 5,000, Creative PEGWorks) and 550 mg of PEBP-alkyne (MW = 6300) were dissolved in 10 mL DMF and degassed by N_2_ for 30 min. Next, 6 mg CuBr (Sigma-Aldrich) and 10 mg *N*,*N*,*N*′,*N*′′,*N*′′-pentamethyldiethylenetriamine (PMDETA, Sigma-Aldrich) were quickly added in a sealed environment under N_2_ protection to initiate the click reaction (Fig. 2). After stirring overnight, a 20 kDa MW dialysis membrane (Spectra/Por) was used to remove unreacted materials with the dialysate replaced five times. The freeze–dried powder was characterized by ^1^H NMR in CDCl_3_ solution and stored at −20 °C.

### Self-assembly of mPEG-bPEI-PEBP in water

The mPEG-bPEI-PEBP 2 mg was dissolved in 0.2 mL THF by sonication. Deionized water (1 mL) was added dropwise and the mixture sonicated for 1 min. The THF was then removed by evaporation overnight, generating an opalescent solution. The resultant mPEG-bPEI-PEBP micelles were characterized by transmission electron microscopy (TEM, JEM-1200EX, 120 kV). The zeta potential and size of the nanoparticles were measured by nanoparticle analyzer (SZ-100, HORIBA).

### Cytotoxicity assay of the effect of mPEG-bPEI-PEBP on Jurkat and K562 cells

Jurkat and K562 cells were seeded at a concentration of 2 × 10^5^ in a 96-well plate. Following overnight incubation, cells were treated with 100 μL of mPEG-bPEI-PEBP medium (RPMI 1640, Corning Inc.) containing approximately 20, 2, 0.2, 0.02, 0.002, 0.0002, 0.00002, and 0.000002 mg mL^−1^ of mPEG-bPEI-PEBP per well. After incubation for another 24 h, the 3-(4,5-dimethylthiazol-2-yl)-2,5-diphenyltetrazolium (MTT, Sigma-Aldrich) assay was used to evaluate cell viability. The cells were centrifuged at 1500 g, the supernatant was removed and 100 μL of MTT solution (0.5 mg mL^−1^ in PRMI 1640 medium) was added for 4 h. The medium was then removed and 100 μL of DMSO was added to each well to completely dissolve the crystals. The absorbance at 570 nm (Thermo Multiskan GO) was recorded, and cytotoxicity was calculated as the percentage of viable cells compared with the blank control. Each data point was represented as a mean ± standard deviation of at least five independent experiments.

### Loading the mPEG-bPEI-PEBP with anti-CD19 CAR lentivirus plasmids

Based on the MTT assay results, mPEG-bPEI-PEBP 200 μg mL^−1^ is weakly cytotoxic to Jurkat and K562 cells (Fig. S1†). Therefore, the 200 μg mL^−1^ was selected as the final working concentration of mPEG-bPEI-PEBP to achieve maximum loading. mPEG-bPEI-PEBP co-polymer 2 mg along with 1, 5 and 20 μg of lentivirus plasmids (CAR : psPAX2 : pMD2.G = 4 : 3 : 1, w/w) were dissolved in 1 mL ddH_2_O and vortexed for 10 min. The mPEG-bPEI-PEBP-DNA mixture was then rocked gently at 4 °C overnight to further improve electrostatic adsorption efficiency. Formation of complexes between the co-polymer and DNA was characterized by mixing 1, 0.5, 0.25, 0.125, 0.063, 0.031, and 0.016 mg mL^−1^ mPEG-bPEI-PEBP with 20 μg mL^−1^ CAR plasmids in an aqueous solution for 1 h, and subjecting the product to electrophoresis in 1% agarose gel. The co-polymer and DNA mixture was prepared freshly just before each use.

### Co-transfection with three plasmids

The mPEG-bPEI-PEBP-plasmids mixture solution was prepared as described above, included 200 μg mL^−1^ mPEG-bPEI-PEBP plus 1, 5 and 20 μg mL^−1^ plasmids. Following vortexing for a few seconds, 200 μL of the mixture was added dropwise to previously prepared 5 × 10^6^ Jurkat cells in 2 mL of medium and seeded in a 6-well plate. The plates were returned to the incubator at 37 °C and 5% CO_2_. Lipofectamine® 2000 commercial reagent (Lipo2000, Thermo Co., Ltd) was used as a positive control.

### Fluorescence imaging and flow cytometry of mPEG-bPEI-PEBP-DNA transfected Jurkat cells and CD19-K562 cells

To further evaluate the transfection and expression effects of mPEG-bPEI-PEBP delivered CAR plasmids, Jurkat cells treated as above were imaged by fluorescence microscopy. The three concentrations (1, 5 and 20 μg mL^−1^) of DNA plasmids with 200 μg mL^−1^ mPEG-bPEI-PEBP treated cells were placed under an inverted microscope and observed in bright field and GFP detection channel at the 48 h time point. Subsequently, the cells were resuspended in PBS and analyzed by flow cytometry (FCM). The optimal transfection concentration of mPEG-bPEI-PEBP was used to treat Jurkat cells for 72 h. The cells were observed by fluorescence microscopy (Olympus IX73) and analyzed by FCM at 24, 48 and 72 h time points. The plates were centrifuged at 800 g for 3 min to precipitate suspended cells before microscopic examination. For GFP detection, the blue excitation channel was selected; for RFP detection, the green excitation channel was used.

### Biologic effects of mPEG-bPEI-PEBP-DNA transfected Jurkat cells on CD19-K562 cells

To further study the biologic effects of mPEG-bPEI-PEBP-DNA transfected Jurkat cells (transfected Jurkat cells), secretion of T-helper type I cytokines, interferon-γ (IFN-γ) and interleukin-2 (IL-2), was measured using commercially available ELISA reagents (Human IL-2 ELISA Kit, Human IFN-γ ELISA Kit, Becton, Dickinson and Company). The impact of transfected Jurkat cells on CD19-K562 cells was also determined by lactate dehydrogenase (LDH) cytotoxicity assay (CytoTox 96® Non-Radioactive Cytotoxicity Assay, Promega). The transfected Jurkat cells (effector Cell, E) and CD19-K562 cells (target Cell, T) were mixed and incubated in a 24-well plate at a density of 10^6^ cells per well and E/T ratios of 2 : 1, 4 : 1 and 8 : 1. For comparison, both Jurkat cells incubated with K562 cells (Jurkat + K562) and CAR-Jurkat cells incubated with K562 cells (CAR-Jurkat + K562) were used.

### Statistical analysis

At least three independent samples were tested in each group, and *t* test was done to perform a statistical. The Statistical Product and Service Solutions software was used for statistical analysis. *P* < 0.05 was considered as statistically significant.

## Results and discussion

### Synthesis of mPEG-bPEI-PEBP co-polymer and self-assembly in water

**Fig. 3:**
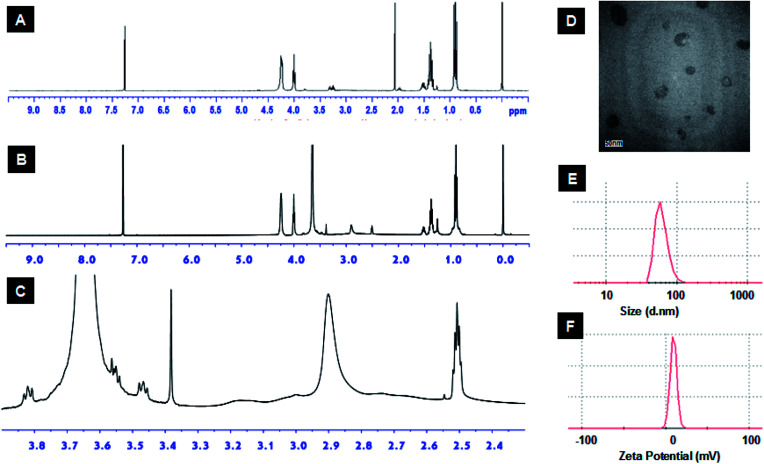
The ^1^H NMR characterization of the synthesized alkyne-PEBP (A) and mPEG-bPEI-PEBP (B) which comprised a branched PEI 2000 at 2.3–3.3 ppm (C); the mPEG-bPEI-PEBP co-polymer self-assemble to form micelles in aqueous solution; they were characterized by transmission electron microscopy (D, scale bar = 50 nm); the micelles have an average diameter of 62.2 ± 27.3 nm (E) and zeta potential of 9.5 mV (F).

The azido functionality was introduced to bPEI *via* reaction with azidoacetic acid as described elsewhere.^26^ The EBP monomer was identified by ^1^H NMR and ^31^P NMR (Fig. S2†), and the following data were obtained, ^1^H NMR (CDCl_3_, ppm): *δ* 0.95 (t, *J* = 7.4 Hz, POCH2CH(*CH2CH3*)2), 1.38 (m, POCH2CH(*CH2CH3*)2), 1.54 (m, 1H, POCH2C*H*), 4.05 (m, POC*H2*CH), 4.21–4.30 (br m, POC*H2*C*H2*OP), ^31^P NMR (CDCl_3_, ppm): *δ* −1.13. Next, alkyne-terminated PEBP was synthesized *via* ring-opening polymerization (ROP), a reaction extensively used to synthesize various biocompatible and degradable polymers including polyesters, polypeptides and polyphosphoesters.^30–32^ The biocompatible organic bicyclical strong guanidine base, TBD, was employed as the catalyst for EBP polymerization. Propargyl alcohol was used as the initiator to introduce alkyne functional groups. The synthesized alkyne-PEBP was purified three times by precipitation from dichloromethane into petroleum ether and vacuum-dried as a colorless transparent liquid with a yield of approximately 87%. The polymer was dissolved in CDCl_3_ and characterized by ^1^H NMR. The characteristic peaks of EBP were observed (Fig. 3A and S3†). The EBP monomers and 2-propynyl alcohol initiator were mixed in a ratio of 29 : 1. The ^1^H NMR results indicated the polymer had 30 repeating units consistent with the target ratio and confirming successful synthesis of the PEBP with alkyne as one of the end groups. PEBP is a biodegradable hydrophobic polymer with excellent biocompatibility.^33,34^ In a report by Zhang and coworkers, 1,8-diazabicyclo[5.4.0]undec-7-ene (DBU) or 1,5,7-triazabicyclo[4.4.0]dec-5-ene (TBD) were used as organocatalysts to promote ROP of cyclic phospholanes effectively.^35,36^

The amphiphilic polymer, mPEG-bPEI-PEBP, was synthesized by conjugating PEBP-alkyne and mPEG-alkyne to the bPEI-azide backbone *via* the highly efficient copper(i)-catalyzed azide–alkyne Huisgen cycloaddition (CuAAC). Copper and other impurities were removed by two days of dialysis. Due to the strong hydrophobic properties of PEBP, the amphiphilic co-polymer spontaneously self-assembled into nanoparticles during dialysis. In fact, formation of a clear opalescent solution during dialysis was observed. This demonstrated successful synthesis of the three-component amphiphilic co-polymer. The characteristic peaks of all three components, mPEG (*δ* 3.35–3.9), bPEI (*δ* 2.3–3.3) and PEBP, were also found in the ^1^H NMR spectrum (Fig. 3B and C). The integration ratios from the ^1^H NMR spectrum indicated that each mole of bPEI (2 kDa), with about 23 monomer units, contained about 188 hydrogen and 27 nitrogen atoms. These were conjugated with approximately one equivalent of mPEG-alkyne (5 kDa) and 2 equivalents of alkyne-PEBP (about 6 kDa) (Fig. S4†). The molecular weight of mPEG-bPEI-PEBP was about 20 kDa. These values were similar to those expected from the results obtained for the added raw materials since PEI containing 2 mmol amines and 0.15 mmol azido groups was conjugated with 0.043 mmol mPEG-alkyne and 0.086 mmol PEBP-alkyne.

The synthesized mPEG-bPEI-PEBP was then dissolved in aqueous solution to allow self-assembly into micelles. Micelle morphology was characterized by TEM, which indicated the presence of monodispersed organic particles with a size of approximately 50 nm (Fig. 3D). The zeta potential and the size of the nanoparticles were measured by nanoparticle analyzer, and found to be 9.5 mV and 62.2 ± 27.3 nm (mean diameter), respectively (Fig. 3E and F). These data showed that the amphiphilic polymer, mPEG-bPEI-PEBP, could self-assemble into nanoparticles in water while retaining its positive electric charge. PEG provides a biocompatible interface with the water phase, and hydrophobic PEBP is an excellent biodegradable polymer. Together, they form an amphiphilic co-polymer capable of wrapping the bPEI cationic polymer inside and self-assembly into nanoparticles to reduce cytotoxic side effects. This biocompatible design can facilitate future *in vivo* applications of this novel nanocarrier.

### Optimization of transfection conditions for mPEG-bPEI-PEBP mediated anti-CD19 CAR production in Jurkat cells

The PEI polymer rich in amine groups displayed a high cationic charge density capable of forming ionic interactions with the phosphate backbone of the DNA. The PEI–DNA complex traversed through the negatively charged cell membrane to deliver the loaded DNA into cells. Here, the genetic material could exert its biological function. DNA-PEI complexes can escape endosomes *via* ‘proton sponge effects’ that promote osmotic swelling leading to disruption of the endosomal membrane.^37^ Previous studies indicated that the molar ratio of PEI nitrogen atoms and DNA phosphate atoms, expressed as the N/P ratio, correlated with the efficiency of DNA transfection.^38,39^ It has also been shown that the N/P ratios, together with an excess of PEI, generate the ‘proton sponge effect’ to breakdown endosomes and lysosomes.^40,41^

**Fig. 4:**
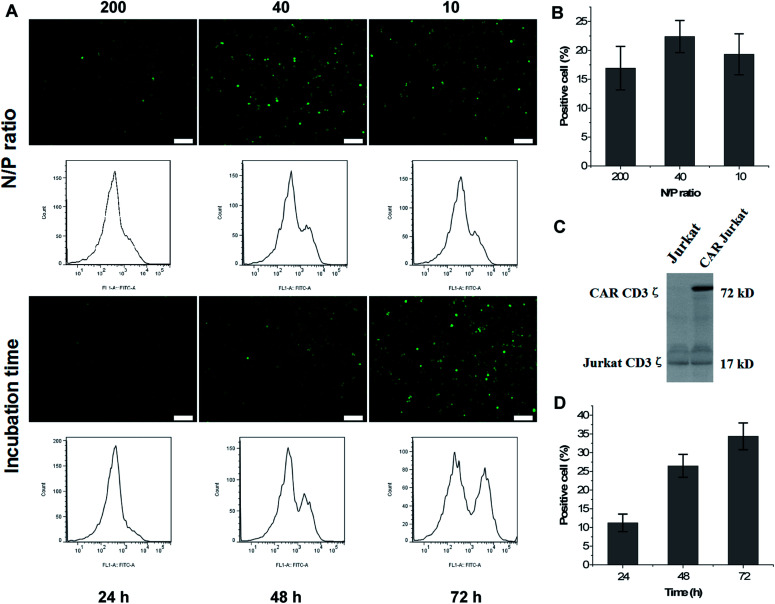
The CAR vector with a GFP reporter gene, and packing plasmids were co-transfected into Jurkat cells at different concentrations and examined by fluorescent microscopy or FCM at 48 or 72 h; the 200 : 1, 40 : 1, and 10 : 1 N/P ratios of lentivirus plasmids-loaded nanocarriers were incubated with Jurkat cells for 48 h (A, N/P ratio); scale bar = 300 μm; (B) the fraction of positive cells in the three treated groups; the mPEG-bPEI-PEBP-DNA mixture (N/P ratio equal 40 : 1) was then incubated with Jurkat cells to study the CAR expression at 24, 48, and 72 h time points (A, incubation time); the FCM results are presented below; (D) the fraction of positive cells at 24, 48, and 72 h time points; (C) the immunoblot assay (anti-human CD3ζ) utilized to detect the CAR expression demonstrates a significant new 72 kDa band.

The formation of a complex between the co-polymer and DNA was characterized by agarose gel electrophoresis. The results demonstrated that most of the DNA (20 μg mL^−1^) formed an effective and stable bond with mPEG-bPEI-PEBP at co-polymer concentrations exceeding 0.125 mg mL^−1^ (Fig. S5†). To further elucidate the biological effects of CAR delivered nanocomposites in Jurkat cells, CAR vectors with the GFP reporter gene and the packaging vectors were co-transfected into Jurkat cells at different concentrations with fluorescent signals detected 48 or 72 h later. Since the synthesized mPEG-bPEI-PEBP with PEI 2000 included 40–50 units of nitrogen, the experiments used lentivirus plasmid concentrations of 1, 5 and 20 μg mL^−1^ (ratio of CAR : psPAX2 : pMD2.G = 4 : 3 : 1, w/w) loaded onto 200 μg mL^−1^ nanocarriers. The N/P ratios were approximately 200 : 1, 40 : 1 and 10 : 1, and transfected DNA was maintained at 0.6, 3, and 12 μg cm^−1^ plate. After 48 h incubation, fluorescence imaging and FCM tests were performed. The results indicated that 200 μg mL^−1^ of mPEG-bPEI-PEBP was capable of transfecting all three concentrations of DNA plasmids into Jurkat cells and express the GFP (Fig. 4A, N/P ratio). The N/P ratio most effective in transfection was 40 : 1 (5 μg mL^−1^ plasmids with 200 μg mL^−1^ of mPEG-bPEI-PEBP), and yielded 22.4 ± 2.79% positive cells (Fig. 4A, N/P ratio 40). The 200 : 1 (16.93 ± 3.75% positive cells, Fig. 4A N/P ratio 200) and 10 : 1 (19.33 ± 3.55% positive cells, Fig. 4A N/P ratio 10) N/P ratios also resulted in GFP expression but with weaker fluorescence than the 40 : 1 N/P ratio (Fig. 4B). Therefore, the N/P ratio was maintained at 40 : 1 in subsequent experiments.

The mPEG-bPEI-PEBP-DNA mixture was added to 6 well-plates and incubated with Jurkat cells to study CAR expression at 24, 48 and 72 h. Both fluorescence microscopy observations and FCM results showed that with increasing incubation time, the fluorescence signal and the fraction of GFP positive cells were also increased (Fig. 4A, incubation time). The fraction of positive cells at 24, 48 and 72 h was 11.23 ± 2.35%, 26.47 ± 3.07% and 34.37 ± 3.58%, respectively (Fig. 4D). The anti-human CD3ζ immunoblot assay was performed to detect CAR expression which was composed of CD19 scfv, 4-1BB and CD3ζ. With this method, a significant new band appeared at 72 kDa, which indicated the expression of the CAR against CD19 antigen (Fig. 4C). These results demonstrated that the co-transfected lentivirus plasmids were effectively delivered and expressed in Jurkat cells. Moreover, as a result of the direct instantaneous transfer, the three-plasmid system including the CAR core plasmid and two packaging plasmids also jointly generated virus particles to stably transfect more cells. This resulted in higher levels of CAR and GFP expression.

Lipofectamine 2000 was employed as the positive control of transfection efficiency. Although the transfection efficiency with lipofectamine was higher than with the co-polymer within the first 12 h, the difference was no longer statistically significant at 72 h time point (Fig. S6†). Importantly, lipofectamine needs to be removed after 6 h of incubation to reduce its cytotoxic side effect, while the mPEG-bPEI-PEBP-DNA complex can be present for a longer time to extend transfection action and achieve results comparable to lipofectamine at 72 h. The excellent cell transfection efficiency of the mPEG-bPEI-PEBP cationic nanocarrier was likely achieved by the ‘proton sponge effect’ of coated PEI. This promotes endosome osmotic swelling and destruction of endosomes as well as helping encapsulated DNA escape into cytoplasm to become available for transcription and expression.^42^

### Biologic effects of mPEG-bPEI-PEBP-DNA transfected Jurkat cells on CD19-K562 cells

**Fig. 5:**
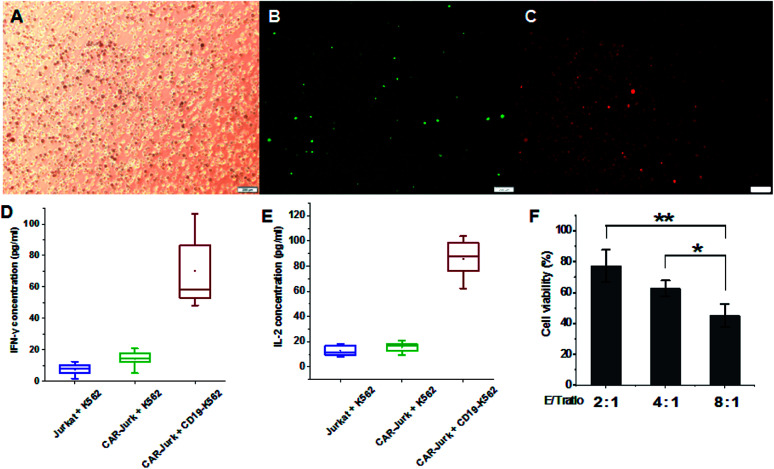
The mPEG-bPEI-PEBP-DNA transfected Jurkat cells were mixed with CD19-K562 cells ((A) bright field panel, (B) green fluorescence panel, (C) red fluorescence panel, scale bar = 200 μm); after incubation for 48 h, the IFN-γ (D) and IL-2 (E) expression was detected by ELISA assay; the cytotoxic effects were measured at different E/T ratios (2 : 1, 4 : 1, 8 : 1) by the LDH assay (F). Data are expressed as mean ± sd. **P < 0.01, *P < 0.05.

To further investigate the effects of mPEG-bPEI-PEBP-DNA transfected Jurkat cells (CAR-Jurkat cells), the target cell K562 expressing antigen CD19, serving as CAR target cells, were incubated with CAR-Jurkat cells (CAR-Jurkat + CD19-K562, Fig. 5 ABC). After 48 h of incubation, secretion of T-helper type I cytokines, IFN-γ and IL-2 was determined by commercial ELISA assay (Fig. 5D and E, S7†). For comparison, Jurkat cells incubated with K562 cells (Jurkat + K562) and CAR-Jurkat cells incubated with K562 cells (CAR-Jurkat + K562) were used as control. The ELISA results showed that secretions of both IFN-γ and IL-2 in the CAR-Jurkat + CD19-K562 group were significantly increased compared with the Jurkat + K562 group and the CAR-Jurkat + K562 group. This finding indicated that Jurkat cells expressing anti-CD19 CAR and T cell activators can stimulate secretion of T cell cytokines by target CD19-K562 cells.

The cytotoxic effect of CAR-Jurkat cells on CD19-K562 was next investigated by LDH release assay. The results indicated that the increase in E/T ratio potentiated cytotoxicity. The E/T ratio of 8 : 1 was associated with higher cell death than the ratios of 4 : 1 and 2 : 1 (Fig. 5F). These findings demonstrated that mPEG-bPEI-PEBP-DNA transfected Jurkat cells express the anti-CD19 CAR functional elements and target the CD19-K562 cells for immune-mediated cell death.

## Conclusions

In summary, a novel three-segment co-polymer, mPEG-bPEI-PEBP, was designed and synthesized by the click reaction. The biocompatible PEBP and mPEG parts of the molecule provide hydrophobic and hydrophilic interfaces, while the PEI provides cationic charge for loading of anti-19 CAR and packaging plasmids. The amphiphilic co-polymer can self-assemble into nanoparticles in water and encapsulate the DNA plasmids to serve as a delivery carrier. The formed nanocomposites can co-transfect plasmids into Jurkat cells and produce anti-CD19 CAR. The transfection N/P ratio and incubation time were optimized to achieve the highest delivery efficiency. Finally, the functions of CAR-Jurkat cells were studied through incubation with CD19-K562 cells. Secretion of both IFN-γ and IL-2 were found to be increased, and cytotoxicity also augmented at a higher E/T ratio.

## Conflicts of interest

There are no conflicts to declare.

## Supplementary Material

RA-008-C8RA02133C-s001
