# Supplementary material for: A novel phosphoester-based cationic co-polymer nanocarrier delivers chimeric antigen receptor plasmid and exhibits anti-tumor effect
Source: RSC Adv. 2018 Apr 19;8(27):14975–82. doi: 10.1039/c8ra02133c (PMC9080078; doi:10.1039/c8ra02133c)
Supplement: RA-008-C8RA02133C-s001 [file RA-008-C8RA02133C-s001.pdf]

Electronic Supplementary Material (ESI) for RSC Advances.
This journal is © The Royal Society of Chemistry 2018

A novel phosphoester-based cationic co-polymer nanocarrier delivers chimeric antigen receptor plasmid and exhibits anti-tumor effect

Jing Fan,[a, b] Qianjun He,[c] Zhaokui Jin,[c] Wei Chen[a,*] and Weiren Huang[a,*]

[a]*Key Laboratory of Medical Reprogramming Technology, Shenzhen Second People's Hospital, First Affiliated Hospital of Shenzhen University, Shenzhen 518039, China*

[b]*Sun Yat-sen University Cancer Center, State Key Laboratory of Oncology in South China, Guangzhou 510060, China*

[c]*Guangdong Key Laboratory for Biomedical Measurements and Ultrasound Imaging, School of Biomedical Engineering, Health Science Center, Shenzhen University, No. 3688 Nanhai Road, Nanshan District, Shenzhen 518060, China*

Corresponding Authors

*Email for W.H. : pony8980@163.com; *email for W.C. : jessie_chenwei@163.com

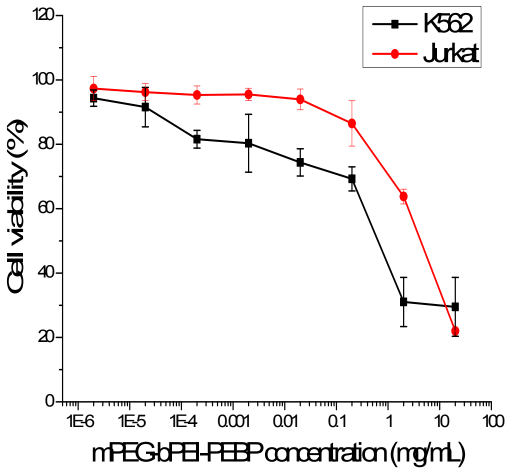

Figure S1. The cytotoxicity assay of mPEG-bPEI-PEBP in Jurkat and K562 cells. The results indicate that 200 μg/mL of mPEG-bPEI-PEBP leads to weak cytotoxicity to Jurkat cells and K562 cells.

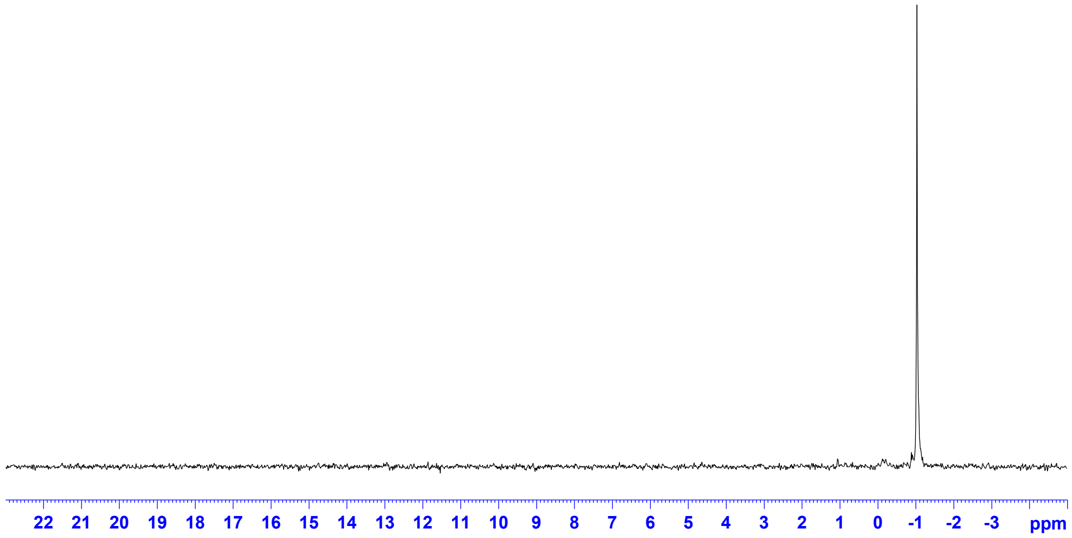

Figure S2. The ^{31}P NMR characterization of the synthesized 2-ethylbutyl phospholane; a single peak was detected at δ -1.13.

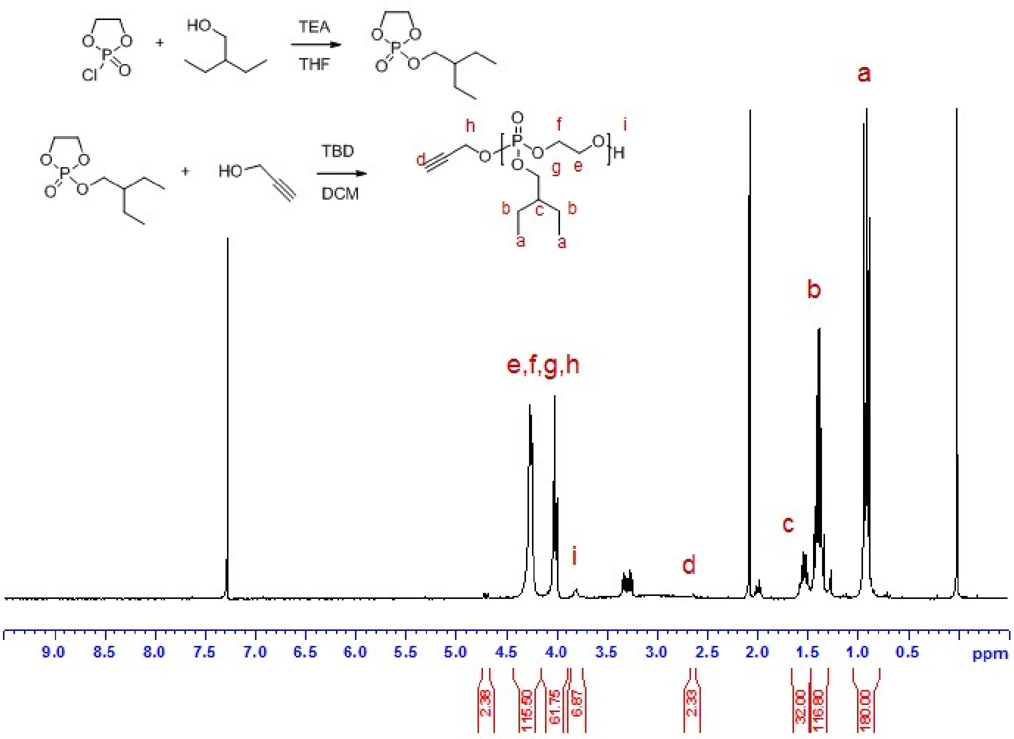

Figure S3. Reactions of synthesis of alkyne-PEBP and ^{1}H NMR characterization of the product. The corresponding characteristic peaks and integration values are presented. TEA, triethylamine; THF, tetrahydrofuran; TBD, 1,5,7-triazabicyclo[4.4.0]dec-5-ene; DCM, dichloromethane.

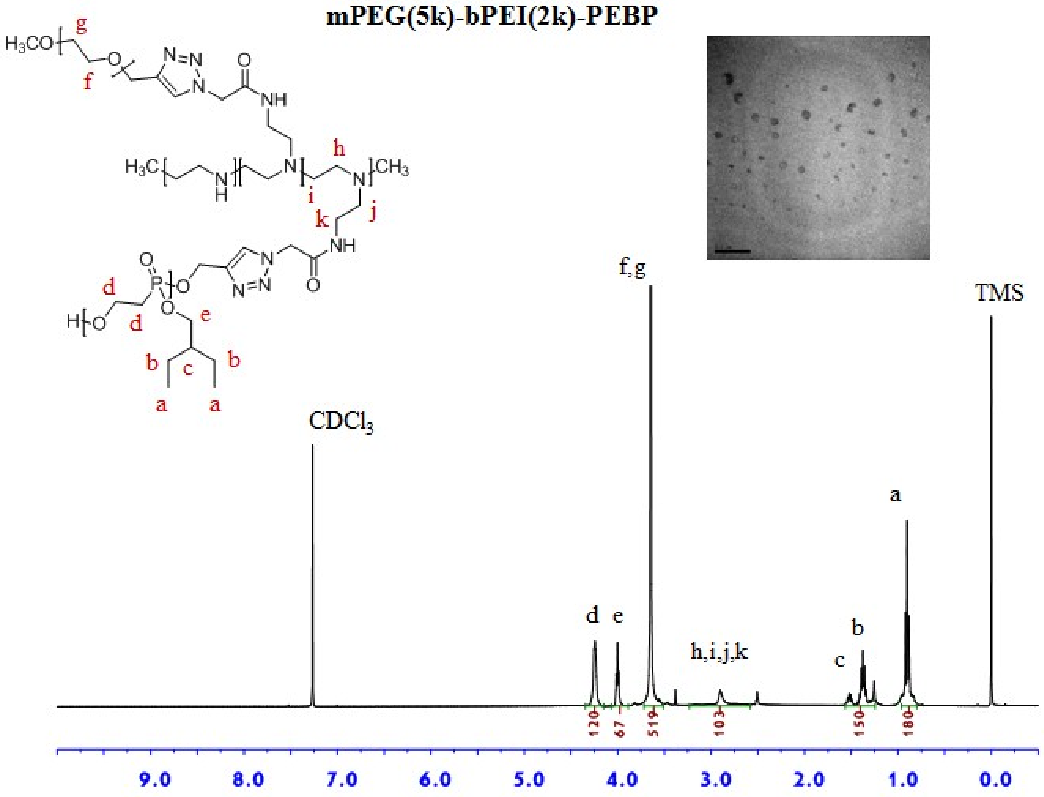

Figure S4. The synthesized mPEG(5k)-bPEI(2k)-PEBP was charaterized by ^{1}H NMR. The corresponding characteristic peaks and integration values are presented. The inset represents a TEM image of co-polymer self-assembled in an aqueous solution (scale bar=200 nm). TMS, tetramethylsilane.

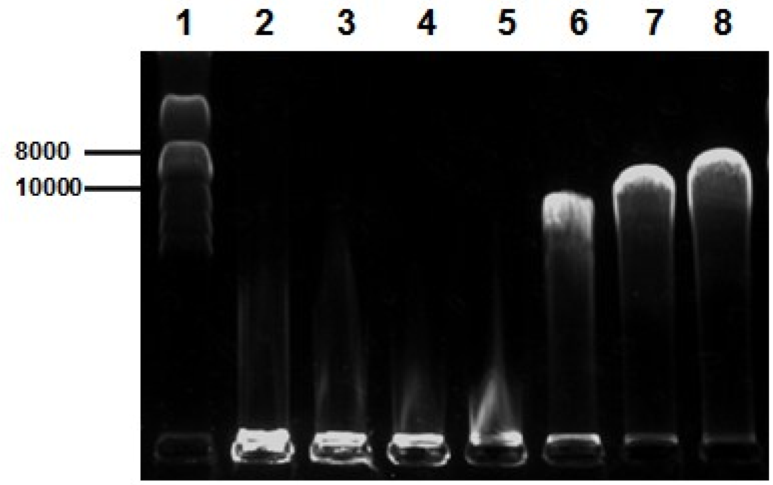

Figure S5. Formation of a complex between the co-polymer and DNA was characterized by agarose gel electrophoresis. Aliquots of 1 (lane 2), 0.5 (lane 3), 0.25 (lane 4), 0.125 (lane 5), 0.063 (lane 6), 0.031 (lane 7), and 0.016 (lane 8) mg/mL mPEG-bPEI-PEBP were mixed with 20 µg/mL CAR plasmids in an aqueous solution and subjected to electrophoresis in 1% agarose gel. Lane 1, DNA molecular weight marker.

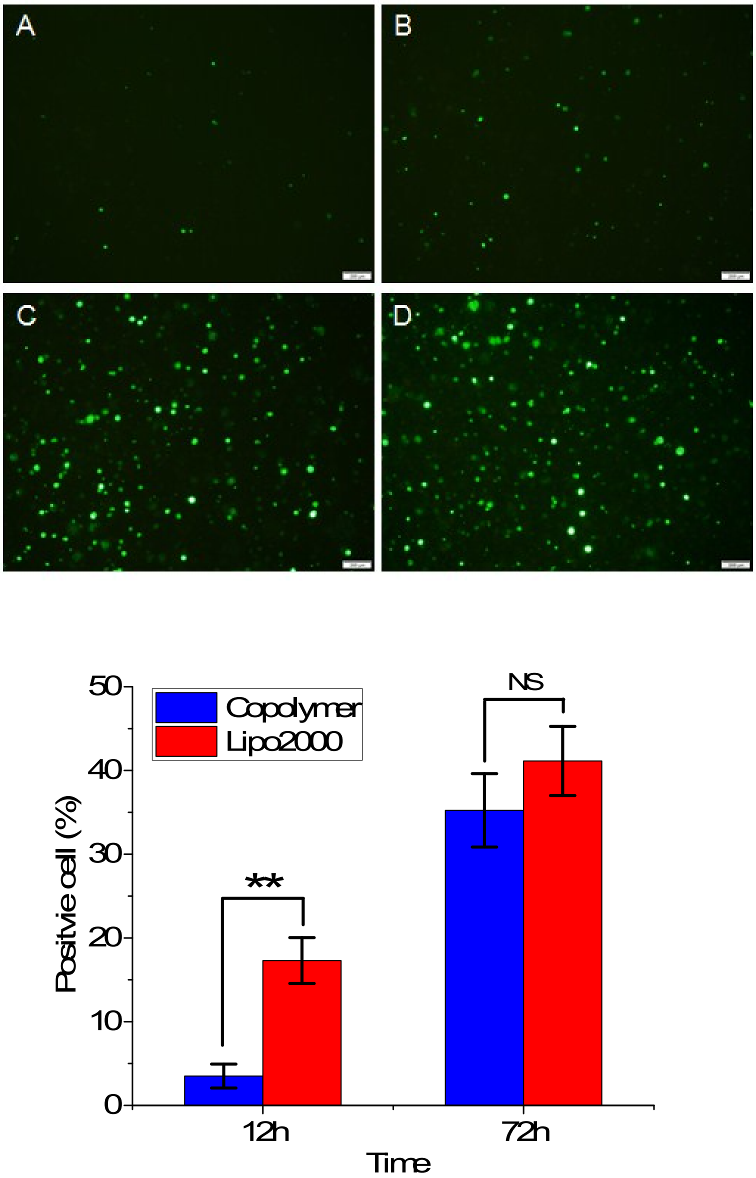

Figure S6. Fluorescent images illustrating the transfection efficiency of the mPEG-bPEI-PEBP co-polymer and lipofectamine 2000 (Lipo2000) at 12 and 72 h after transfection. A, the co-polymer group at 12 h; B, the Lipo2000 group at 12 h; C, the co-polymer group at 72 h; D, the Lipo2000 group at 72 h; E, Quantitative results. Data are expressed as mean ± sd. **$P < 0.01$; NS, not significant.

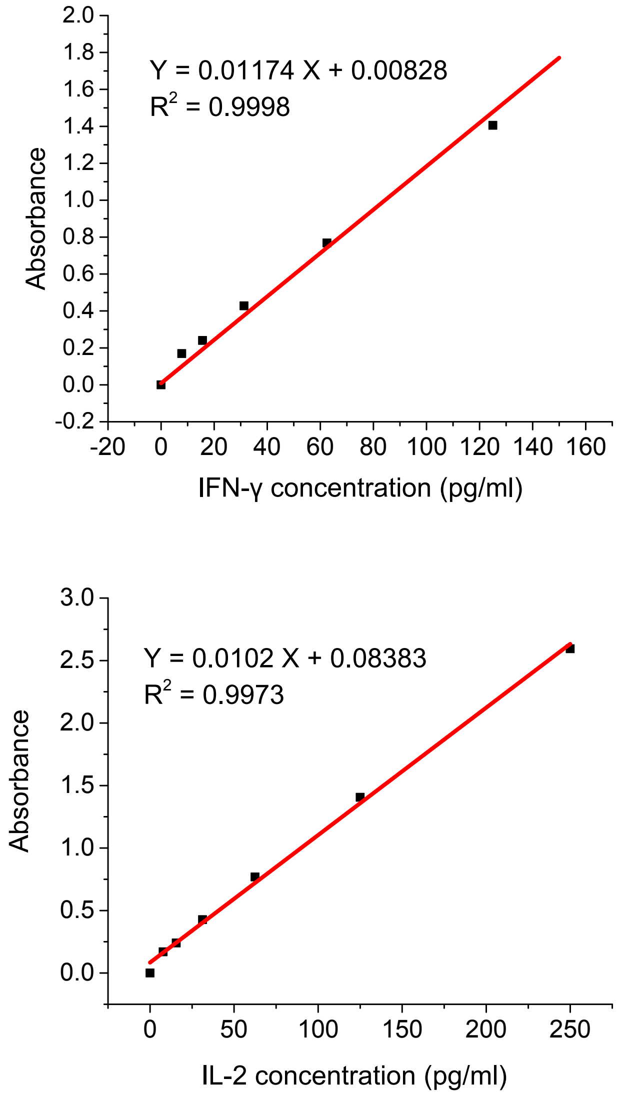

Figure S7. The standard curves of IFN-γ and IL-2 obtained by the ELISA method.
